# Zinc Status and Occurrence of Thyroid Cancer: Systematic Review and Meta-Analysis

**DOI:** 10.3390/nu17172820

**Published:** 2025-08-29

**Authors:** Aline Alves Soares, Yasmin Guerreiro Nagashima, Grasiela Piuvezam, Camila Xavier Alves, Kleyton Santos de Medeiros, Márcia Marília Gomes Dantas Lopes, Jose Brandao-Neto

**Affiliations:** 1 Postgraduate Program in Health Sciences, Federal University of Rio Grande do Norte, Natal 59078-970, RN, Brazil; aaline.alves@hotmail.com (A.A.S.); yasmin_nagashima@yahoo.com.br (Y.G.N.); brandao-neto@live.com (J.B.-N.); 2 Department of Nutrition, Liga Contra o Câncer, Natal 59062-000, RN, Brazil; 3 Postgraduate Program in Public Health, Federal University of Rio Grande do Norte, Natal 59078-970, RN, Brazil; gpiuvezam@yahoo.com.br; 4 Systematic Review and Meta-Analysis Laboratory (Lab-SYS), Federal University of Rio Grande do Norte, Natal 59078-970, RN, Brazil; 5 Monsenhor Walfredo Gurgel Hospital, Natal 59015-000, RN, Brazil; camila_xavieralves@yahoo.com.br; 6 Institute of Education, Research and Innovation, Liga Contra o Câncer, Natal 59062-000, RN, Brazil; kleyton_medeiros@hotmail.com; 7 Department of Nutrition, Postgraduate Program in Sciences Applied to Women’s Health, Federal University of Rio Grande do Norte, Natal 59078-970, RN, Brazil

**Keywords:** literature review, zinc, trace elements, thyroid gland neoplasms

## Abstract

**Background and Objective**: Thyroid cancer (TC) represents the most common group of endocrine tumors, and its incidence has increased over the last four decades. The imbalance of trace elements, such as zinc (Zn), has been investigated due to the thyroid’s sensitivity to these elements. Zn is essential for thyroid hormone action and may be involved in the pathogenesis of TC. This systematic review and meta-analysis aim to contribute to the discussion on the association between low serum Zn concentrations and the occurrence of TC. **Materials and Methods**: The search was carried out in the PubMed/MEDLINE, Scopus, Embase, LILACS and Web of Science databases, including observational studies published until December 2024. The primary outcome was low serum Zn concentration and the occurrence of TC. Three independent reviewers selected the studies and extracted the data from the original publications. The risk of bias was assessed using the Newcastle–Ottawa Quality Assessment Scale. Data analysis was performed using R software (V.4.3.1), and heterogeneity was calculated using the *I*^2^ statistic, with results based on random effects models. **Results**: A total of 10 studies (*n* = 7 case–control and *n* = 3 cross-sectional) with sample sizes ranging from 44 to 294 individuals were included. The results indicated that serum Zn levels were not significantly lower in patients with TC compared with healthy controls (mean difference: −251.77; 95% confidence interval: −699.09, 195.54; *I*^2^ = 100%, very low certainty). **Conclusions**: Further investigations, including rigorously designed observational studies with representative samples and improved control of potential confounding variables are indispensable.

## 1. Introduction

Thyroid cancer (TC) originates from the disordered proliferation of follicular epithelial cells and is one of the most prevalent neoplasms among endocrine and neuroendocrine tumors [1]. Its incidence has increased significantly in recent decades, and projections suggest that, by 2030, TC could become the fourth most common type of cancer [2].

The histological classification of TC encompasses three main categories: differentiated carcinomas, which include papillary, follicular, and oncocytic subtypes; medullary carcinoma, often associated with multiple endocrine neoplasia type 2 syndromes; and anaplastic carcinoma, generally derived from differentiated forms and characterized by high mortality [3]. Among these, papillary carcinoma represents the most common subtype, corresponding to approximately 84% of cases [4]. The main cause related to the development of papillary carcinoma is exposure to ionizing radiation during childhood [5,6].

The increase in the incidence of TC can be explained by the greater availability and accuracy of diagnostic techniques. However, several authors argue that this technological evolution does not, by itself, justify the observed increase, with the additional influence of environmental factors, lifestyle habits and comorbidities being likely [7,8,9].

Studies indicate that dietary aspects also impact the risk of developing TC. Among the dietary factors identified, the Western dietary pattern [10] and the high intake of nitrates and nitrites present in processed meats, which can produce potentially carcinogenic compounds [11], stand out. Such evidence reinforces the role of diet in maintaining thyroid health.

The thyroid gland’s sensitivity to trace elements has fueled research into its imbalance, highlighting zinc (Zn) as a possible marker in the diagnosis and prognosis of thyroid disorders, including cancer. Studies indicate an association between reduced Zn levels and changes in thyroid function [12,13,14,15,16,17,18]. However, knowledge about its homeostasis is still relatively new. Zn balance depends on strict control mechanisms, primarily via intestinal excretion. In cases of very low intake, the body draws on reserves present in metabolically active tissues, such as the liver and kidneys [19,20].

Despite regulatory mechanisms, Zn absorption does not adapt efficiently to the body’s needs [21], making certain groups, such as children, pregnant women, and individuals with infections or diarrhea, more vulnerable to deficiency [20,21,22]. Physiologically, Zn is essential for thyroid function, influencing the synthesis and regulation of TRH, TSH, T3, and T4. Its deficiency can compromise this hormone production [23]. Furthermore, Zn acts as a cofactor for superoxide dismutase (SOD), contributing to antioxidant defense and protection against cellular damage associated with cancer development [24,25].

Given its wide range of biological functions, Zn deficiency is associated with several physiological impairments, including impaired body growth, immunological dysfunctions, reproductive alterations and disorders in neurobehavioral development [20].

In the context of the relationship between Zn and TC, research has shown that patients with TC have significantly lower serum Zn levels compared with controls. Although some studies suggest a possible link between Zn deficiency and increased risk for TC, the findings remain inconclusive [13,14,16,26].

Given this scenario, it is important to further investigate the biological mechanisms involved in Zn deficiency and its possible relationship with thyroid carcinogenesis. Therefore, it is important to contribute to the scientific literature by conducting a systematic review and meta-analysis, with the aim of identifying and synthesizing the evidence available in primary studies that evaluate the association between low serum Zn concentration and the occurrence of TC in adults.

## 2. Materials and Methods

The systematic review and meta-analysis were conducted following the Meta-Analysis of Observational Studies in Epidemiology (MOOSE) guidelines [27], according to the checklist criteria Preferred Reporting Items for Systematic Reviews and Meta-analysis (PRISMA) [28,29]. The protocol was registered in the International Prospective Register of Systematic Reviews (PROSPERO) under number CRD42023463747.

### 2.1. Search Strategy

The databases PubMed/MEDLINE, Embase, Scopus, LILACS and Web of Science were used to identify observational studies on low serum Zn and the occurrence of TC published until December 2024. A manual search of the records was performed in the references of the articles included in the review.

Medical Subject Headings (MESH) descriptors were used, and the following search terms were selected: ((Zinc) AND (Thyroid Neoplasm OR Neoplasm, Thyroid OR Thyroid Carcinoma OR Carcinoma, Thyroid OR Cancer of Thyroid OR Thyroid Cancer OR Cancer, Thyroid OR Thyroid Adenoma OR Adenoma, Thyroid) AND (Observational Study)). Based on the terms described, and using Boolean operators (AND and OR), search equations adapted to the specific syntax of each database were constructed, as shown in Table S1 (Supplementary Materials). The search strategy was validated by retrieving previously read articles that met the inclusion criteria and were on the list of research related to the strategy used. In addition, the librarian also participated in this stage of developing the search strategy.

### 2.2. Inclusion Criteria

Observational cohort, case–control and cross-sectional studies that evaluated serum Zn concentrations (in serum, plasma and whole blood) and their association with the occurrence of TC in individuals over 18 years of age in an apparently healthy population (in the control groups for case–control studies) were included. There was no restriction regarding the publication period, and articles written in any language were considered. In addition, the selected studies should present the calculable mean and standard deviation (SD) of serum Zn concentrations.

### 2.3. Exclusion Criteria

Case reports, meeting abstracts, review articles and commentaries were not considered.

### 2.4. Study Selection Process

After applying the search strategy, all identified publications were exported to the Rayyan^®^ software (https://www.rayyan.ai/). Initially, automatic duplicate detection was performed. Then, the researcher (AAS) removed duplicate studies and reviews. In the next step, two researchers (AAS and YGN) independently examined the titles and abstracts to identify the studies that met the eligibility criteria. In cases of divergence, the final decision on inclusion was made with the participation of a third researcher (CXA). The selected articles were then evaluated in full by AAS and YGN, also independently, to confirm their eligibility. Throughout the review process, the reasons for exclusion of the studies were carefully recorded.

### 2.5. Data Extraction

The Cochrane tool [30], a standardized electronic spreadsheet, was developed for data extraction. Two reviewers (AAS and YGN) performed this process independently for each included study. In case of inconsistencies, the discrepancies were discussed and resolved with the help of a third reviewer (CXA). When missing, suppressed or incomplete data were identified, the reviewers contacted the authors or co-authors of the respective studies by means of e-mail communication. Additionally, the supplementary documents related to the studies were also reviewed. If it was not possible to obtain the necessary information, these studies were addressed in the discussion section and excluded from the analysis.

The extracted data included information such as the name of the first author; year of publication; country/continent; sample size; gender and age of participants; number of participants in the case group (if case–control study); number of participants in the control group (if case–control study); type of study; follow-up period; eligibility criteria; serum Zn levels (in patients with TC and control subjects); type of serum Zn sample (serum, plasma, or whole blood); Zn measurement methods; quantitative method of variable analysis; and the mean and SD of serum Zn levels. Similarly, the mean differences (MD) and 95% confidence interval (CI) for the occurrence of TC were extracted.

### 2.6. Risk of Bias and Quality Assessment

The included studies were assessed for methodological quality, with the aim of evaluating the risk of bias, using the Newcastle–Ottawa Quality Assessment Scale (NOS) tool [31]. Two researchers (AAS and YGN) performed this assessment independently.

The NOS tool covers eight criteria organized into three main domains: choice of study groups, comparability of groups, and exposures or outcomes of interest. Each criterion is assigned one point or one star, with the exception of “Comparability,” which can be assigned up to two stars. High-quality studies were assigned a rating of at least six stars; moderate-quality studies were assigned four to five stars; and low-quality studies were assigned less than four stars.

### 2.7. Data Synthesis

The *I*^2^ test was used to assess heterogeneity between studies, with values below 25% indicating low heterogeneity, between 25% and 50% indicating moderate heterogeneity, and above 50% indicating high heterogeneity [30]. The meta-analysis was conducted using R software, version 4.3.1. Low serum Zn values and the occurrence of TC were presented by SD and 95% CI, using random effects models.

## 3. Results

### 3.1. Survey and Selection of Studies

**Figure 1:**
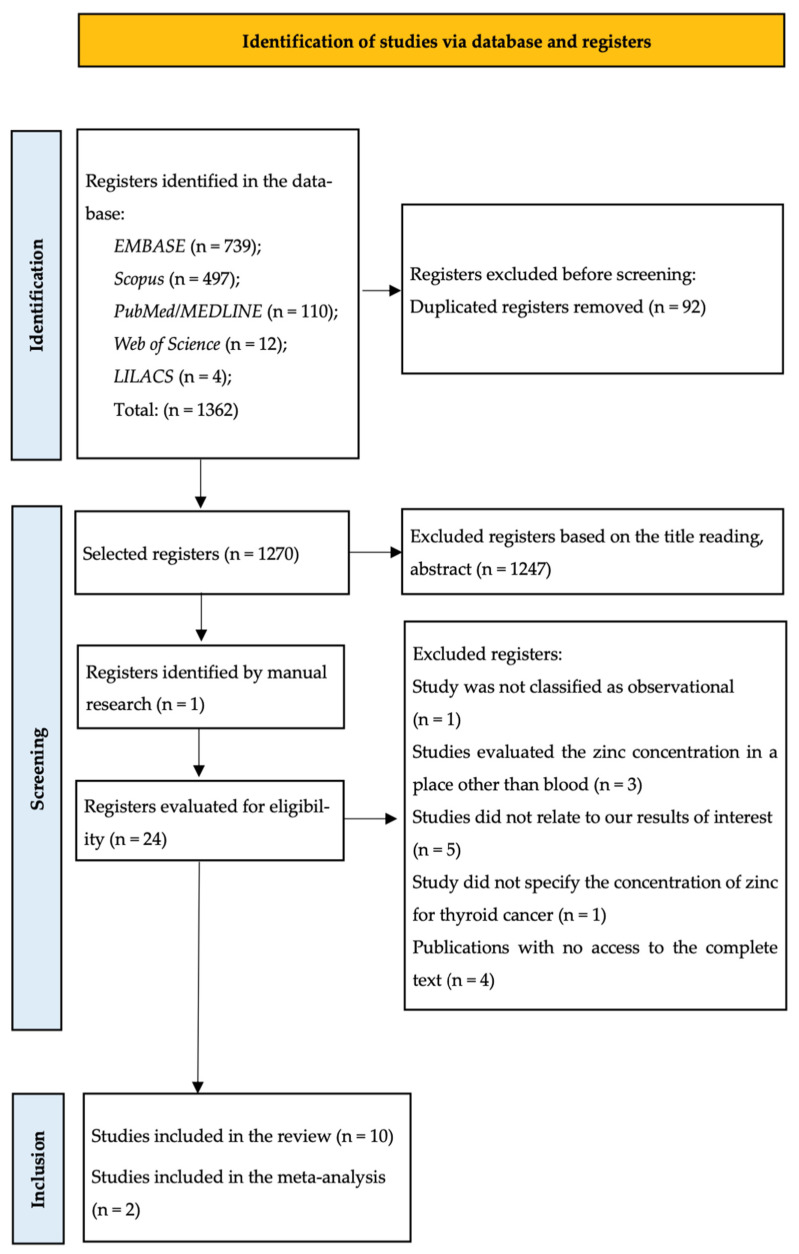
Flowchart of the article selection process.

Of the 1362 records obtained from the research databases, 10 articles were selected and evaluated, of which 7 were case–control studies [13,14,16,17,18,26,32] and 3 were cross-sectional studies [12,33,34]. All articles were published between 2004 and 2024. Four studies for which we did not have access to the document or contact with the authors were excluded [35,36,37,38]. Although 10 studies were included in the systematic review, only 2 met the criteria for meta-analysis due to data availability and methodological compatibility [16,18]. Figure 1 presents the diagram with the study selection process.

### 3.2. Characteristics of the Studies

The included studies were conducted in nine countries and on one continent: Serbia [13], China [12], Pakistan [16], Turkey [17], Korea [34], Poland [32], Kuwait [18], Iran [26,33] and Africa [14]. The number of participants ranged from 44 to 294 individuals. Regarding the type of biological sample for serum Zn analysis, six studies used serum samples [16,17,18,26,32,33], two used plasma [14,15] and two used whole blood [12,34].

The duration of the studies varied between 7 and 29 months; however, six of them did not specify the follow-up period. The participants were adults and elderly, and in two studies the sample was composed exclusively of women [32,34].

Regarding the type of TC, it was identified as unspecified TC [12,26], papillary [13,14,34], medullary [17,33] and variable types of TC [16,18,32]. In seven studies, TC was confirmed by histopathological examination [13,14,16,17,18,32,34]. In one study, confirmation was performed by clinical laboratory investigation by fine needle aspiration [26]. In another study, the benignity and malignancy of the thyroid nodule were evaluated by ultrasound [12]. Finally, in one study, the method of TC diagnosis was not detailed [33].

Regarding the method used to measure Zn concentrations, three studies applied inductively coupled plasma mass spectrometry (ICP-MS) [13,26,34], while three others [16,18,32] applied atomic absorption spectrometry (AAS). These two methods stood out as the most frequently applied for Zn measurement in the analyzed studies. The main characteristics of the included studies are summarized in Table 1.

### 3.3. Meta-Analysis of the Studies

Two studies investigated the relationship between low serum Zn levels and the occurrence of TC [16 and 18]. To enable the meta-analysis, it was necessary to standardize the measures of variability associated with serum Zn concentrations. One of the studies [16] used the relative SD, which was converted to the sample standard deviation—a measure also adopted by the other study [18]. In addition, serum Zn values were converted to μg/dL, ensuring equivalence between studies. In the study carried out by Al-Sayer H et al. [18], serum Zn levels were presented separately for two types of TC (papillary and follicular); therefore, the mean serum Zn concentrations of patients in the case group were used as the variable of interest.

**Figure 2:**
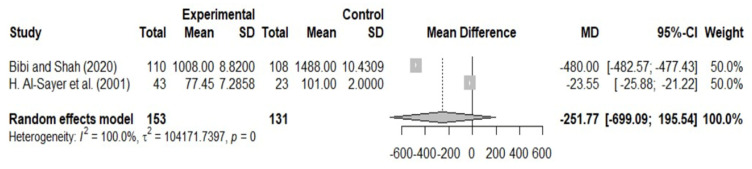
Forest plot of the association between low serum zinc and the occurrence of thyroid cancer [16,18].

However, the analysis indicated that the association between low serum Zn levels and the occurrence of TC was not significant when compared to the control group (MD = −251.77; 95% CI: −699.09–195.54), as illustrated in Figure 2. Additionally, heterogeneity was assessed, and the Higgins *I*^2^ index indicated 100% heterogeneity (*p*-value = 0).

### 3.4. Risk of Bias Analysis

All studies scored higher than 6 on the NOS, indicating high quality (Table 1). The domains with the highest risk of bias were exposure determination and outcome of interest not present at baseline.

## 4. Discussion

This systematic review and meta-analysis indicated that in individuals with TC, serum Zn concentrations were not significantly lower than in healthy controls. Consistent with these findings, the case–control study conducted by Rezaei M et al. [26] also did not identify a significant association between serum Zn levels and the occurrence of TC. Furthermore, the study by Emami A et al. [33], analyzing the micronutrient status in Iranian patients with medullary thyroid cancer (MTC) before thyroidectomy, demonstrated that low serum Zn levels did not represent a risk factor for the development of MTC.

Corroborating these results, the meta-analysis performed by Gumulec J et al. [39], which included data from three studies involving 131 cases and 93 controls, also did not identify a significant difference in serum Zn levels in individuals with TC.

On the other hand, the findings of Stojsavljević A et al. [13] revealed that the mean Zn concentration (1613 ng/g) in blood samples from TC patients was significantly lower (*p* < 0.05) compared to the control group (5147 ng/g). This result may play an important role from a clinical point of view, especially with regard to the diagnosis and screening of the disease.

These findings from this study point to altered levels of Zn in blood samples with the disease when compared to controls, suggesting that the relationship between serum Zn and TC still remains controversial [13], reinforcing the need for more in-depth investigations on the subject.

In this review, the inconsistency of data regarding the association between low serum Zn concentration and the occurrence of TC can be attributed to the heterogeneity among the studies analyzed. This heterogeneity is due to several factors, such as the limited number of studies included in the meta-analysis, the small sample size, the short follow-up period, the diversity of the types of TC evaluated, the variation in the methods used to measure Zn, and the different geographical origins of the participants—with most studies conducted in Asian populations, two in European populations, and one in an African population.

This trend has also been observed in other types of cancer. For example, a meta-analysis involving 36 studies of breast cancer patients identified a significant association between decreased serum Zn concentrations and the risk of ongoing breast cancer or recurrences. However, due to the high heterogeneity among the studies, the authors recommended that more primary studies be conducted [40]. Similarly, a meta-analysis analyzing 52 studies of prostate cancer indicated that serum Zn levels may play an important role in the development of the disease. However, as observed in our study, the findings remain inconsistent, suggesting the need for more comprehensive and well-designed investigations [41].

In another larger meta-analysis [39], which evaluated the associations between different types of cancer and serum Zn levels based on the analysis of 144 articles, a reduction in serum Zn concentrations was identified in patients with lung, head and neck, and breast cancer. However, due to the high heterogeneity between the studies, the data did not allow definitive conclusions.

Overall, research findings on the role of Zn suggest relevance not only for the diagnosis but also for the treatment of cancer. As an example, a systematic review involving 23 studies suggested that Zn may be beneficial in preventing oral side effects associated with radiotherapy [42].

The present study has some strengths, including updating previous evidence from Gumulec J et al. [39] and exploring the association between low serum Zn concentrations and the occurrence of TC in adults. Furthermore, the selected studies were limited to the analysis of Zn concentrations in serum, plasma and whole blood. The evidence suggests that, although these measurements may have limitations in terms of validity and reliability for identifying mild to moderate Zn deficiencies in individuals, the measurement of Zn in serum or plasma, for example, is useful in assessing Zn status at the population level [43,44]. It is the most frequently used biomarker to measure Zn levels, and it is also the only one with accepted cut-off values, used clinically for the identification of deficiency in individuals [45].

Regarding the potential of this study, the use of ICP-MS, a sensitive method that allows the measurement of metals at low concentrations, stands out [46]. Despite the variation in the analytical techniques used to measure Zn levels among the studies included in our systematic review and meta-analysis, it is relevant to highlight that the majority used the aforementioned technique.

Furthermore, Zn acts as a cofactor for the copper (Cu) and Zn superoxide dismutase (SOD) enzyme in the body, and its imbalance may result in oxidative stress. Thus, serum Zn deficiency may indicate an intense activation of the antioxidant response during TC. In general, oxidative stress associated with Zn deficiency is related to damage to DNA integrity, favoring cellular transformation and, consequently, the development of neoplasia [47]. Such evidence suggests that reduced serum Zn may function as a biomarker for TC. As an example, the study conducted by Poo JL et al. [48] indicated that an altered Cu:Zn ratio may be a useful biomarker for digestive tract cancers.

Furthermore, this study used meta-analysis, an important tool for quantitative data synthesis, allowing the integration of studies with similar methodologies and results. The assessment of the risk of bias of the included studies, carried out using the NOS tool, indicated that all of them were classified as having a low risk of bias.

However, in addition to the observed heterogeneity, other limitations should be considered. Although only studies that evaluated Zn concentrations in serum, plasma or whole blood were included, such measurements are not entirely comparable, mainly due to the different collection and separation methods used [49]. Furthermore, the studies used non-standardized techniques for the quantification of serum Zn (Table 1), and no information was reported on the different tumor stages of the disease. Variations were also identified in the units of measurement adopted for serum Zn (Table 1), as well as in the reference values used, such as 10.7–17.7 μmol/L [12], 70–127 mg/dL [33], and 80–120 μg/L [34].

Additionally, the reduced serum Zn concentrations observed in the included studies may be attributed to different conditions, representing a normal physiological response, and not necessarily indicating a Zn-deficient status. In inflammatory contexts, such as cancer, serum Zn levels decrease, possibly due to redistribution of the mineral from plasma to the liver. This condition should be considered with caution before Zn level is used as a biomarker for cancer [49].

Studies on metabolic balance indicate that failures of the homeostatic response to restore Zn balance are driven by the loss of a small mobilizable pool of Zn, representing less than 2% of the total body, mainly in blood plasma. However, the majority of Zn in the body, located in muscle tissue, bones and organs (2–3 g in adult men), is highly conserved, even under severe dietary restrictions. This reinforces the importance of detecting the level of this element in another type of biological sample, such as tissues [45].

As a limiting factor, it is important to consider the inverse relationship between Cu and Zn. A diet excessively rich in Zn can compromise Cu absorption, while high concentrations of Cu can, in turn, reduce Zn absorption [50,51]. These interactions can influence the results obtained in patients, based on changes in the Cu/Zn ratio. In this sense, the proportion between these two elements can have a broader meaning than just the isolated values of serum Zn levels, due to the complex interaction between these essential micronutrients [43].

Recent studies also highlight the influence of dietary factors on the risk of TC. In a case–control study, Xia M et al. (2023) suggested that diets rich in refined grains, sodium, added sugars, and saturated fats may be associated with an increased risk of developing TC [52].

These findings are in line with the study by Sangsefidi ZS et al. (2019) [10], which demonstrated that adherence to a Western dietary pattern, characterized by foods rich in fats, sugars and ultra-processed foods, significantly increased the risk of developing differentiated thyroid cancer (DTC). In contrast, the same study did not identify a significant association between other dietary patterns (traditional, transitional or healthy) and the risk of DTC, indicating that not all diets have the same risk potential for the development of the disease [10].

The influence of diet on thyroid health is evident, as a single nutrient or dietary intake can influence the risk of TC [25]. The lack of dietary diversity in the Western pattern may result in insufficient consumption of Zn-rich food sources, such as red meat, seafood, and dairy products, which may compromise thyroid function. Furthermore, dietary factors, such as the presence of phytate, may inhibit Zn absorption [49].

In this context, it is essential to understand the relationship between dietary aspects, nutrient intake and frequency of food consumption in the development of TC. Thus, strategies aimed at diversifying and modifying the diet are essential to optimize both the intake and absorption of Zn, promoting balanced diets rich in essential trace elements, such as Zn.

### Important Deviations from the Published Protocol

We report no significant deviations from the published protocol [53].

## 5. Conclusions

Current evidence suggests that low serum Zn concentrations (measured in plasma, serum or whole blood) do not present a statistically significant correlation with the occurrence of TC. To better elucidate this relationship, more robust research is needed, including well-designed observational studies with larger samples, stratification by tumor stage and rigorous control of confounding variables. It is noteworthy that the present study focused exclusively on the analysis of Zn concentrations in blood and may not be sufficiently representative. Therefore, the evaluation of this trace element in other biological samples, such as tissues, may be relevant, especially in scenarios of failures in the Zn homeostatic response.

Maintaining Zn homeostasis requires understanding endogenous losses, dietary influences, and health impacts of deficiency. Therefore, additional studies are essential to fill existing gaps and improve nutritional strategies associated with this trace element.

## Figures and Tables

**Table 1:** Characteristics of the studies included in the systematic review and meta-analysis.

First Author	Year of Publication	Country/Continent	Type of Study	*n* (Cases/ Controls)	Mean (SD) Age (Cases/Controls)	TC Type	TC Serum Zn (Mean ± SD)	Controls Serum Zn (Mean ± SD)	Zn Measurement Method	Total Score (NOS)
Ma Q [12]	2024	China	CS	294/-	46.62/NA	TC	13.9 μmol/L	14.2 μmol/L	The automatic biochemical analyzer	8
Stojsavljević A [13]	2021	Serbia	CC	66/65	40/40	PTC	1613 ng/g ± 376.46	5147 ng/g ± 1130	ICP-MS	7
Kazi Tani LS [14]	2021	Africa	CC	46/50	40/43	PTC	942.1 μg/dL	1027.9 μg/dL	TXRF	9
Bibi K and Shah MH [16]	2020	Pakistan	CC	110/108	48.5/48.1	ATC/MTC/ FTC/PTC	1008 μg/dL ± 0.875	1488 μg/dL ± 0.701 *	AAS	9
Rezaei M [26]	2019	Iran	CC	11/33	37/39.39	TC	15.6 μg/g ± 5.2	66.7 μg/g ± 49.9	ICP-MS	7
Emami A [33]	2017	Iran	CS	40/40	40—49/NA	MTC	89.45 μg/dL ± 4.70	94.13 μg/dL ± 10.65	enzyme immunosorbent	7
Baltaci [17]	2017	Türkiye	CC	30/20	50 ± 5/ND	MTC	Male: 65.5 μg/dL ± 6.8 Woman: 66.2 μg/dL ± 7.2	Male: 98.5 μg/dL ± 11.4 Female: 92.7 μg/dL ± 10.2	ICS-AES	8
Chung [34]	2016	Korea	CS	92/-	43.6/NA	PTC	60.19 μg/dL ± 6.58	-	ICP-MS	6
Przybylik-Mazurek [32]	2011	Poland	CC	38/20	PTC: 51.6 FTC: 52.5/37.7	PTC/FTC	PTC: 12.2 μM/L ± 2.1 FTC: 12.0 μM/L ± 2.1	12.5 μM/L ± 2.8	AAS	6
Al-Sayer H [18]	2004	Kuwait	CC	43/23	33.4/18—53	PTC/FTC	PTC: 822 μg/L ± 92 FTC: 727 μg/L ± 113	1010 μg/L ± 20	AAS	8

Abbreviations: CC: case–control; CS: cross-sectional; TC: thyroid cancer; PTC: papillary thyroid cancer; ATC: anaplastic thyroid cancer; MTC: medullary thyroid cancer; FTC: follicular thyroid cancer; ICP-MS: inductively coupled plasma mass spectrometry; AAS: atomic absorption spectrometry; ICS-AES: inductively coupled plasma atomic emission spectrometry; TXRF: total reflection X-ray fluorescence; NA: not applicable; ND: not available; SD: standard deviation; NOS: Newcastle–Ottawa Quality Assessment Scale; * Relative Standard Deviation (RSD).

## Data Availability

All data of this study is available from the corresponding author on reasonable request.

## Supplementary Materials

The following supporting information can be downloaded at https://www.mdpi.com/article/10.3390/nu17172820/s1, Table S1: Search strings used for searching Pubmed, Embase, Lilacs, Scopus and Web of Science.

## References

[1] Boufraqech M., Patel D., Xiong Y., Kebebew E. (2013). Diagnosis of thyroid cancer: State of the art. Expert Opin. Med. Diagn..

[2] Rahib L., Smith B.D., Aizenberg R., Rosenzweig A.B., Fleshman J.M., Matrisian L.M. (2014). Projecting Cancer Incidence and Deaths to 2030: The Unexpected Burden of Thyroid, Liver, and Pancreas Cancers in the United States. Cancer Res..

[3] Chen D.W., Lang B.H.H., McLeod D.S.A., Newbold K. (2023). Haymart, MR Thyroid cancer. Lancet.

[4] Boucai L., Zafereo M. (2024). Cabanillas, ME Thyroid Cancer: A Review. JAMA.

[5] Ron E., Lubin J.H., Shore R.E., Mabuchi K., Modan B., Pottern L.M., Schneider A.B., Tucker M.A., Boice J.D. (1995). Thyroid cancer after exposure to external radiation: A pooled analysis of seven studies. Radiat. Res..

[6] Morton L.M., Karyadi D.M., Stewart C., Bogdanova T.I., Dawson E.T., Steinberg M.K., Dai J., Hartley S.W., Schonfeld S.J., Sampson J.N. (2021). Radiation-related genomic profile of papillary thyroid carcinoma after the Chernobyl accident. Science.

[7] Nettore I.C., Colao A. (2018). Macchia PENutritional Environmental Factors in Thyroid Carcinogenesis. Int. J. Environ. Res. Public Health.

[8] Cho Y.A., Kim J. (2014). Thyroid cancer risk and smoking status: A meta-analysis. Cancer Causes Control..

[9] Pappa T., Alevizaki M. (2014). Obesity and thyroid cancer: A clinical update. Thyroid.

[10] Sangsefidi Z.S., Ghafouri-Taleghani F., Zakavi S.R., Norouzy A., Kashanifar R., Pourbaferani R., Safarian M., Hosseinzadeh M. (2019). Major dietary patterns and differentiated thyroid cancer. Clin. Nutr. ESPEN.

[11] Neto V., Leitão C., Estrela M., Fardilha M., Herdeiro M.T., Nunes A. (2024). The influence of nutrition in nodular thyroid pathology: A systematic review. Crit. Rev. Food Sci. Nutr..

[12] Ma Q., Li Y., Yu G., Liu S., Jiang Y., Duan H., Wang D., He Y., Chen X., Yao N. (2024). Sex-Specific Associations of Five Serum Essential Metal Elements with Thyroid Nodules in Euthyroid Adults: A Cross-sectional Study. Biol. Trace Elem. Res..

[13] Stojsavljević A., Rovčanin B., Jagodić J., Krstić Đ., Paunović I., Gavrović-Jankulović M., Manojlović D. (2021). Alteration of Trace Elements in Multinodular Goiter, Thyroid Adenoma, and Thyroid Cancer. Biol. Trace Elem. Res..

[14] Kazi Tani L.S., Gourlan A.T., Dennouni-Medjati N., Telouk P., Dali-Sahi M., Harek Y., Sun Q., Hackler J., Belhadj M., Schomburg L. (2021). Copper Isotopes and Copper to Zinc Ratio as Possible Biomarkers for Thyroid Cancer. Front. Med..

[15] Mehl S., Sun Q., Görlich C.L., Hackler J., Kopp J.F., Renko K., Mittag J., Schwerdtle T., Schomburg L. (2020). Cross-sectional analysis of trace element status in thyroid disease. J. Trace Elem. Med. Biol..

[16] Bibi K., Shah M.H. (2020). Appraisal of Metal Imbalances in the Blood of Thyroid Cancer Patients in Comparison with Healthy Subjects. Biol. Trace Elem. Res..

[17] Baltaci A.K., Dundar T.K., Aksoy F., Mogulkoc R. (2017). Changes in the Serum Levels of Trace Elements Before and After the Operation in Thyroid Cancer Patients. Biol. Trace Elem. Res..

[18] Al-Sayer H., Mathew T.C., Asfar S., Khourshed M., Al-Bader A., Behbehani A., Dashti H. (2004). Serum changes in trace elements during thyroid cancers. Mol. Cell. Biochem..

[19] Maywald M., Wessels I., Rink L. (2017). Zinc Signals and Immunity. Int. J. Mol. Sci..

[20] King J.C., Brown K.H., Gibson R.S., Krebs N.F., Lowe N.M., Siekmann J.H., Raiten D.J. (2015). Biomarkers of Nutrition for Development (BOND)-Zinc, Review. J. Nutr..

[21] Fung E.B., Ritchie L.D., Woodhouse L.R., Roehl R., King J. (1997). CZinc absorption in women during pregnancy lactation: Alongitudinal study. Am. J. Clin. Nutr..

[22] Wessells K.R., Brown K.H. (2012). Estimating the global prevalence of zinc deficiency: Results based on zinc availability in national food supplies and the prevalence of stunting. PLoS ONE.

[23] Severo J.S., Morais J.B.S., Freitas T.E.C., Andrade A.L.P., Feitosa M.M., Fontenelle L.C., de Oliveira A.R.S., Cruz K.J.C., Marreiro D. (2019). The Role of Zinc in Thyroid Hormones Metabolism. Int. J. Vitam. Nutr. Res..

[24] Yao G., Wang Z., Xie R., Zhanghuang C., Yan B. (2024). Trace element zinc metabolism and its relation to tumors. Front. Endocrinol..

[25] Nguyen L.T.D., Gunathilake M., Lee J., Kim J. (2023). Association between dietary habits and incident thyroid cancer: A prospective cohort study. Front. Nutr..

[26] Rezaei M., Javadmoosavi S.Y., Mansouri B., Azadi N.A., Mehrpour O., Nakhaee S. (2019). Thyroid dysfunction: How concentration of toxic and essential elements contribute to risk of hypothyroidism, hyperthyroidism, and thyroid cancer. Environ. Sci. Pollut. Res. Int..

[27] Stroup D.F., Berlin J.A., Morton S.C., Olkin I., Williamson G.D., Rennie D., Moher D., Becker B.J., Sipe T.A., Thacker S.B. (2000). Meta-analysis of observational studies in epidemiology: Aproposal for reporting Meta-analysis Of Observational Studies in Epidemiology (MOOSE) group. JAMA.

[28] Liberati A., Altman D.G., Tetzlaff J., Mulrow C., Gøtzsche P.C., Ioannidis J.P., Clarke M., Devereaux P.J., Kleijnen J., Moher D. (2009). The PRISMA statement for reporting systematic reviews and meta-analyses of studies that evaluate healthcare interventions: Explanation and elaboration. BMJ.

[29] Moher D., Liberati A., Tetzlaff J., Altman D.G., PRISMA Group (2009). Preferred reporting items for systematic reviews and meta-analyses: The PRISMA statement. PLoS Med..

[30] Higgins J.P.T., Thomas J., Chandler J., Cumpston M., Li T., Page M.J., Welch V.A. (2019). Cochrane Handbook for Systematic Reviews of Interventions [Online].

[31] Wells G.A., Shea B., O’Connell D., Peterson J., Welch V., Losos M., Tugwell P. (2000). The Newcastle-Ottawa Scale (NOS) for Assessing the Quality of Nonrandomized Studies in Meta-Analyses [Online].

[32] Przybylik-Mazurek E., Zagrodzki P., Kuźniarz-Rymarz S., Hubalewska-Dydejczyk A. (2011). Thyroid disorders-assessments of trace elements, clinical, and laboratory parameters. Biol. Trace Elem. Res..

[33] Emami A., Nazem M.R., Shekarriz R., Hedayati M. (2017). Micronutrient status (calcium, zinc, vitamins D and E) in patients with medullary thyroid carcinoma: A cross-sectional study. Nutrition.

[34] Chung H.K., Nam J.S., Ah C.W., Lee Y.S., Kim K.R. (2016). Some Elements in Thyroid Tissue are Associated with More Advanced Stage of Thyroid Cancer in Korean Women. Biol. Trace Elem. Res..

[35] Ma Q., Li Y., Yu G., Liu S., Jiang Y., Duan H., Wang D., He Y., Yao N., Wan H. (2023). Single and combined associations of serum metal with thyroid nodules: A cross-sectional study. Rev. Cuba. Med..

[36] Farrokhi Yekta R., Arefi Oskouie A., Taheri S., Daneshyar H. (2019). The evaluation of serum micro and trace elements in patients with papillary thyroid carcinoma and multinodular goiter. Trace Elem. Electrolytes.

[37] Nasiroglu Imga N., Berker D., Baba S., Kücükler K., Navdar Başaran M., Dağlar G., Guler S. (2017). The role of serum zinc and copper levels in predicting malignancy in differentiated thyroid cancers. Trace Elem. Electrolytes.

[38] Azcano G.A., Susana M., Luís Manuel F., Vladimir G.Y. (1985). Analysis multivariate laboratory data en he diagnosis of neoplasms malignant: VII. Rev. Cuba. Med..

[39] Gumulec J., Masarik M., Adam V., Eckschlager T., Provaznik I., Kizek R. (2014). Serum and tissue zinc in epithelial malignancies: A meta-analysis. PLoS ONE.

[40] Jouybari L., Kiani F., Akbari A., Sanagoo A., Sayehmiri F., Aaseth J., Chartrand M.S., Sayehmiri K., Chirumbolo S., Bjørklund G. (2019). A meta-analysis of zinc levels in breast cancer. J. Trace Elem. Med. Biol..

[41] Shahrokhi Nejad S., Golzari Z., Zangiabadian M., Salehi Amniyeh Khozani A.A., Ebrahimi R., Nejadghaderi S.A., Aletaha A. (2024). The association between zinc and prostate cancer development: A systematic review and meta-analysis. PLoS ONE.

[42] Hoppe C., Kutschan S., Dörfler J., Büntzel J., Büntzel J., Huebner J. (2021). Zinc as a complementary treatment for cancer patients: A systematic review. Clin. Exp. Med..

[43] Gillespie S., Kevin J., Mason J. (1991). Controlling iron deficiency. ACC/SCN State-of-the-Art Series Nutrition Policy Discussion Paper.

[44] Nestel P., Nalubola R. (2000). Manual for wheat flour fortification with iron. Part 1: Guidelines for the Development, Implementation, Monitoring, and Evaluation of a Program for Wheat Flour Fortification with Iron.

[45] Ceballos-Rasgado M., Brazier A.K.M., Gupta S., Moran V.H., Pierella E., Fekete K., Lowe N.M. (2025). Methods of Assessment of Zinc Status in Humans: An Updated Review and Meta-analysis. Nutr. Rev..

[46] Sudhakar P., Latha P., Reddy P.V., Sudhakar P., Latha P., Reddy P.V. (2016). Chapter 17—Analytical Techniques. Phenotyping Crop Plants for Physiological and Biochemical Traits.

[47] Pal A., Chatterjee N., Aaqib M., Isha S., Aninda R., Vincenzo D., Mauro T., Gianluca R., Kalyan R., Rosanna G. (2024). Serum zinc status of patients with colorectal cancer: A systematic review and meta-analysis. J. Trace Elem. Miner..

[48] Poo J.L., Romero R.R., Robles J.A., Montemayor A.C., Isoard F., Estanes A., Uribe M. (1997). Diagnostic value of the copper/zinc ratio in digestive cancer: A case control study. Arch. Med. Res..

[49] Brown K.H., Rivera J.A., Bhutta Z., Gibson R.S., King J.C., Lönnerdal B., Ruel M.T., Sandtröm B., Wasantwisut E., International Zinc Nutrition Consultative Group (IZiNCG) (2004). International Zinc Nutrition Consultative Group (IZiNCG) technical document #1. Assessment of the risk of zinc deficiency in populations and options for its control. Food Nutr. Bull..

[50] Kaslow J.E. (2011). Copper/Zinc Imbalance.

[51] Balay K.S., Hawthorne K.M., Hicks P.D., Chen Z., Griffin I.J., Abrams S.A. (2012). Low Zinc Status and Absorption Exist in Infants with Jejunostomies or Ileostomies Which Persist after Intestinal Repair. Nutrients.

[52] Xia M., Zang J., Wang Z., Wang J., Wu Y., Liu M., Shi Z., Song Q., Cui X., Jia X. (2023). Thyroid cancer and its associations with dietary quality in a 1:1 matched case-control study. Br. J. Nutr..

[53] Soares A.A., Nagashima Y.G., Alves C.X., de Medeiros K.S., Lopes M.M.G.D., Brandão-Neto J. (2024). Zinc and thyroid cancer: A systematic review and meta-analysis protocol. PLoS ONE.

