# Supplementary material for: Zinc Status and Occurrence of Thyroid Cancer: Systematic Review and Meta-Analysis
Source: Nutrients. 2025 Aug 29;17(17):2820. doi: 10.3390/nu17172820 (PMC12430485; doi:10.3390/nu17172820)
Supplement: Supplementary file 1 [file nutrients-17-02820-s001.zip › nutrients-3728978-supplementary.pdf]

Supplementary File

Table S1. Search strings used for searching Pubmed, Embase, Scopus, Lilacs, Scopus and Web of Science.

DATABASE	DATE	SEARCH STRATEGY
PUBMED	**12/21/2024**	(((Zinc[Mesh]) OR (Blood[Mesh]) OR (Serum[Mesh]) OR (Serum*) OR (Blood Serum*) OR (Plasma[Mesh]) OR (Plasma*) OR (Blood Plasm*) OR (Plasm*, Blood) OR (Fresh Frozen Plasm*) OR (Frozen Plasm*, Fresh) OR (Plasm*, Fresh Frozen)) AND ((Thyroid Neoplasms [Mesh]) OR (Thyroid Neoplasm) OR (Neoplasm*, Thyroid) OR (Carcinoma*, Thyroid) OR (Thyroid Carcinoma*) OR (Thyroid Cancer*) OR (Cancer*, Thyroid) OR (Cancer* of the Thyroid) OR (Adenoma*, Thyroid) OR (Thyroid Adenoma*)) AND (("observational study":it)
EMBASE	**12/21/2024**	('zinc'/exp) AND ('thyroid neoplasms' OR (thyroid AND neoplasm) OR (neoplasm*, AND thyroid) OR (carcinoma*, AND thyroid) OR (thyroid AND carcinoma*) OR (thyroid AND cancer*) OR (cancer*, AND thyroid) OR (cancer* AND of AND the AND thyroid) OR (adenoma*, AND thyroid) OR (thyroid AND adenoma*)) AND [embase]/lim
LILACS	**12/21/2024**	((zinc)) AND ((thyroid neoplasms) OR (thyroid neoplasm) OR (neoplasm*, thyroid) OR (carcinoma*, thyroid) OR (thyroid carcinoma*) OR (thyroid cancer*) OR (cancer*, thyroid) OR (cancer* of the thyroid) OR (adenoma*, thyroid) OR (thyroid adenoma*)) AND (db:("LILACS"))
SCOPUS	**12/21/2024**	(("zinc") OR ("zinc content") OR ("Zn") OR ("blood") OR ("blood drop") OR ("blood pool") OR ("blood product") OR ("human blood") OR ("human peripheral blood") OR ("peripheral blood") OR ("sanguis") OR ("tissue blood") OR ("whole blood")) OR (("serum") OR ("blood serum") OR ("concentrated serum") OR ("human serum") OR ("serum preparation")) OR (("plasma") OR ("blood plasma") OR ("human plasma") OR ("plasm") OR ("plasma preparation") OR ("plasma, blood")) AND (("thyroid tumor") OR ("neoplasm of thyroid gland") OR ("neoplastic thyroid") OR ("neoplastic thyroid gland") OR ("thyroid gland neoplasia") OR ("thyroid gland neoplasm") OR ("thyroid gland tumor") OR ("thyroid gland tumour") OR ("thyroid neoplasia") OR ("thyroid neoplasm") OR ("thyroid neoplasms") OR ("thyroid tumorigenesis") OR ("thyroid tumour") OR ("thyroidal tumor") OR ("thyroidal tumour") OR ("tumor of thyroid") OR ("tumor of thyroid gland") OR ("tumor, thyroid gland") OR ("tumour of thyroid") OR ("tumour of thyroid gland") OR ("tumour, thyroid gland")) AND (("observational study":it))
WEB OF SCIENCE	**12/21/2024**	(((((((((((ALL=(Zinc)) OR ALL=(Blood)) OR ALL=(serum*)) OR ALL=(Blood Serum*)) OR ALL=(Plasma*)) OR ALL=(Blood Plasm*)) OR ALL=(Plasm*, Blood)) OR ALL=(Fresh Frozen Plasm*)) OR ALL=(Frozen Plasm*, Fresh)) OR ALL=(Plasm*, Fresh Frozen))) AND (((((((((ALL=(Thyroid Neoplasms)) OR ALL=(Thyroid Neoplasm)) OR ALL=(Neoplasm*, Thyroid)) OR ALL=(Carcinoma*, Thyroid)) OR ALL=(Thyroid Carcinoma*)) OR ALL=(Thyroid Cancer*)) OR ALL=(Cancer*, Thyroid)) OR ALL=(Cancer*, Thyroid)) OR ALL=(Adenoma*, Thyroid)) OR ALL=(Thyroid Adenoma*))) AND ALL=((("observational study":it)))
